# Association between atrial fibrillation and gastrointestinal bleeding: pathophysiology, risk stratification, and management – a narrative review

**DOI:** 10.1097/MS9.0000000000004806

**Published:** 2026-03-03

**Authors:** Anmol Mohan, Abdullah Sultany, Syeda Bisma Fatima, Zim Warda Hasan, Muhammad Danish Butt, Aliaa H. J Alkhazender, Priyanka Mohan Lal, Hasibullah Aminpoor, Hasiba Karimi, Vikash Kumar, Usha Tejwaney, Sarwan Kumar

**Affiliations:** a Department of Internal Medicine, Carle Foundation Hospital, Urbana, IL, USA; b Department of Medicine, Robert Packer Hospital/Guthrie, PA, USA; c Department of Internal Medicine, Karachi Medical and Dental College, Karachi, Pakistan; d Department of Internal Medicine, Clinical and Translational Science, School of Medicine, West Virginia University, Morgantown, WV, USA; e Department of Internal Medicine, CMH Lahore Medical College, Lahore, Pakistan; f Department of Internal Medicine, The Islamic University of Gaza, Gaza, Palestine; g Department of Internal Medicine, Ziauddin University, Karachi, Pakistan; h Faculty of Medicine, Kabul University of Medical Sciences, Kabul, Afghanistan; i Faculty of Medicine, Bezmialem Vakif University, Istanbul, Turkey; j Department of Gastroenterology, The Brooklyn Hospital Center, Brooklyn, New York, USA; k Department of Internal Oncology, Valley Health System, Ridgewood, New Jersey, USA; l Department of Internal Medicine, Wayne State University, Detroit, Michigan, USA

**Keywords:** anticoagulation, atrial fibrillation, gastrointestinal bleeding, multidisciplinary management

## Abstract

Atrial fibrillation (AF) is the most common sustained cardiac arrhythmia, significantly increasing the risk of thromboembolic events, necessitating anticoagulation therapy. However, anticoagulation, particularly with novel oral anticoagulants, elevates the risk of gastrointestinal bleeding (GIB), creating a clinical dilemma in managing AF patients. This narrative review explores the pathophysiology linking AF and GIB, emphasizing the hypercoagulable state in AF and the mucosal damage caused by anticoagulants. Epidemiological data reveal that GIB incidence in AF patients ranges from 1.32% to 5.4% annually, with risk factors including older age, prior GIB, and concomitant antiplatelet use. Risk stratification tools such as CHA_2_DS_2_-VASc and HAS-BLED aid in balancing thromboembolic and bleeding risks, though their predictive performance remains modest. Comparative studies highlight that rivaroxaban carries a higher GIB risk, while apixaban offers a safer profile. Management strategies include proton pump inhibitors for prophylaxis, endoscopic interventions for acute bleeding, and individualized decisions on resuming anticoagulation post-GIB, typically within 7–30 days. Emerging research on the gut microbiome’s role in AF pathogenesis suggests potential novel therapeutic avenues. A multidisciplinary approach involving cardiologists, gastroenterologists, and hematologists is essential to optimize outcomes. Future directions include developing safer anticoagulants, refining risk prediction models, and exploring microbiome-targeted therapies.

## Introduction

Atrial fibrillation (AF) is the most common sustained cardiac arrhythmia, with increasing prevalence as populations age^[^1,2,3^]^. It presents with a wide spectrum of symptoms and severity, categorized by persistence and duration^[^1,2^]^. AF is associated with serious adverse outcomes, including stroke, thromboembolic events, and heart failure^[^2^]^. Management focuses on preventing thromboemboli, controlling ventricular response, restoring sinus rhythm, and maintaining it^[^1^]^. Treatment options include antiarrhythmic drugs, radiofrequency ablation, and device therapy^[^1^]^. AF is frequently encountered in perioperative settings, requiring anesthesiologists to maintain hemodynamics and prevent complications^[^4^]^. Recent research has revealed specific molecular pathways underlying AF pathogenesis, driving the development of personalized diagnostic tools and mechanism-based therapies^[^3^]^. A holistic approach, including lifestyle changes, pharmaceutical and nutraceutical therapy, substrate-based ablation, and neuromodulation, may offer novel strategies for AF management^[^3^]^. Stroke prevention through anticoagulation is crucial in AF management^[^5,6^]^. Traditionally, vitamin K antagonists (VKAs) were the primary anticoagulants used^[^7^]^. However, direct oral anticoagulants (DOACs), previously referred to as novel oral anticoagulants (NOACs), have emerged as effective alternatives with more predictable pharmacokinetics and a lower risk of intracranial hemorrhage (ICH) compared to VKAs^[^7,8^]^. The choice of anticoagulation therapy should be individualized based on stroke and bleeding risk assessments^[^5,6^]^. Other treatment options for AF include cardioversion, ablation, and rate or rhythm control approaches^[^5^]^. As AF prevalence increases with age and poses a significant public health burden, understanding its management is essential for healthcare providers across various care settings^[^5,7^]^.

HIGHLIGHTS

- This paper explores mechanisms connecting atrial fibrillation (AF) and gastrointestinal bleeding, emphasizing anticoagulation effects and vascular integrity.
- This review provides strategies for balancing thromboembolic prevention with bleeding risk in AF patients.
- This paper offers insights relevant to cardiologists, gastroenterologists, and internists managing anticoagulated patients.

Gastrointestinal bleeding (GIB) is a significant complication of anticoagulation therapy^[^9^]^. Although early reports suggested a uniformly increased risk of major GIB with NOACs compared to warfarin, more recent evidence highlights a more nuanced picture: bleeding risk varies by specific agent and by anatomical site of bleeding [upper vs lower gastrointestinal (GI)]^[^9^]^. For example, apixaban has consistently demonstrated a more favorable GI safety profile, whereas rivaroxaban is associated with numerically higher GIB rates—though this difference is often not statistically significant^[^9^]^. Upper GIB (UGIB) is more commonly reported with both warfarin and certain DOACs, whereas lower GIB tends to occur at similar rates across agents^[^9^]^. Managing GIB in anticoagulated AF patients requires a multidisciplinary approach, including endoscopy, initial resuscitation, and consideration of anticoagulant reversal in severe cases^[^10^]^. The decision to resume anticoagulation after a bleeding event must balance the risks of thromboembolism and recurrent bleeding^[^10^]^. Physicians should assess individual stroke and bleeding risks to determine appropriate thromboprophylaxis for AF patients^[^6^]^. Given the clinical dilemma of resuming anticoagulation after a bleeding event, a multidisciplinary approach is required, incorporating resuscitation, endoscopic management, and individualized risk assessment for thromboembolism and recurrent bleeding. These considerations underscore the importance of agent selection and bleeding site assessment in guiding clinical decision-making. Understanding the complex interplay between AF, anticoagulation, and GIB is essential for optimizing patient outcomes and guiding evidence-based therapeutic decisions in diverse clinical settings.

Recent advances in pharmacogenomics have further personalized anticoagulant therapy in AF patients, with genetic polymorphisms in CYP2C9 and VKORC1 influencing warfarin metabolism and dosing requirements^[^11^]^. This precision medicine approach may reduce bleeding complications and improve therapeutic outcomes^[^12^]^. Studies have also suggested a link between AF and GI tract disorders beyond bleeding complications. Evidence shows that AF is more common in individuals with celiac disease and inflammatory bowel disease (IBD), possibly due to shared inflammatory pathways and immune-mediated mechanisms^[^13^]^. This highlights the importance of a systems-based approach to AF management, recognizing the role of systemic inflammation and gut-derived factors in arrhythmogenesis. We confirm that no AI tools were utilized in the conduct or writing of this manuscript, in accordance with the TITAN checklist^[^14^]^.

## Methods – literature search strategy

**Figure 1:**
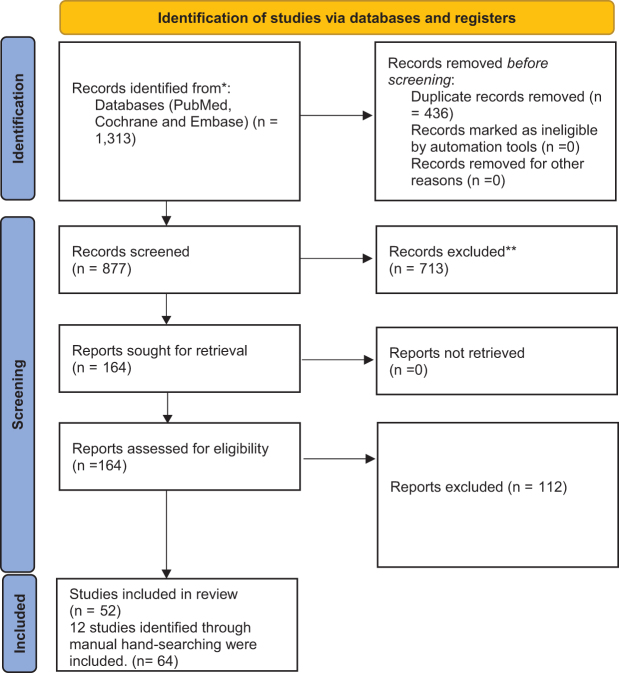
PRISMA chart.

A comprehensive literature search was undertaken to identify studies examining the association between AF, anticoagulant therapy, and GIB. The electronic databases – PubMed, Embase, and the Cochrane Library – were searched systematically for articles published between January 2000 and March 2024. The last search was performed on 15 March 2024. Only human studies published in English were included. The PRISMA chart visually summarizes this in Fig. 1.

To ensure methodological transparency, one full Boolean search string was provided for each database.

### PubMed example search string

(“Atrial Fibrillation”[MeSH] OR “atrial fibrillation”[Title/Abstract] OR “AF”[Title/Abstract]) AND (“Anticoagulants”[MeSH] OR “oral anticoagulants” OR “vitamin K antagonist” OR “warfarin” OR “NOAC” OR “DOAC” OR “direct oral anticoagulants”) AND (“Gastrointestinal Hemorrhage”[MeSH] OR “gastrointestinal bleeding” OR “GI hemorrhage” OR “GI bleeding”).

### Embase example search string

(“atrial fibrillation”/exp OR “atrial fibrillation”:ti,ab OR “AF”:ti,ab) AND (“oral anticoagulant agent”/exp OR warfarin:ti,ab OR “vitamin k antagonist”:ti,ab OR noac:ti,ab OR doac:ti,ab) AND (“gastrointestinal hemorrhage”/exp OR “gastrointestinal bleeding”:ti,ab OR “gi hemorrhage”:ti,ab OR “gi bleeding”:ti,ab).

### Cochrane Library example search string

(“atrial fibrillation” OR “AF”) AND (“oral anticoagulants” OR “NOAC” OR “DOAC” OR “direct oral anticoagulants” OR “warfarin”) AND (“gastrointestinal bleeding” OR “GI hemorrhage” OR “GI bleeding”).

Reference lists of relevant reviews and clinical guidelines were also hand-searched to identify any additional studies.

No formal risk-of-bias or methodological quality appraisal was performed. This limitation is acknowledged explicitly, as recommended by the reviewer.

## Epidemiology and clinical significance

AF is a prevalent cardiovascular condition that increases significantly with age. Studies show that the overall prevalence of AF is less than 1% but rises to 17.8% in those aged 85 and above^[^15,16^]^. The incidence rate also increases with age, from 1.1 per 1000 person-years in the 55–59 age-group to 20.7 in the 80–84 age-group^[^15^]^. AF is more common in men and white individuals^[^17^]^. The prevalence of AF is projected to increase substantially due to population aging, with estimates suggesting a 2.5-fold rise by 2050 in the United States^[^18^]^. AF is associated with significant comorbidities, including heart failure, hypertension, and cerebrovascular disease^[^17^]^. Despite a slight decline over time, mortality rates following AF diagnosis remain high, with 25% of patients dying within 1 year^[^17^]^.

GIB is a significant complication in AF patients on anticoagulation therapy. The incidence of GIB in AF patients ranges from 1.32% to 5.4% per year^[^19,20^]^. Rivaroxaban was associated with a higher rate of GIB compared to warfarin (3.61 vs. 2.60 events/100 patient-years)^[^21^]^. However, aspirin use was linked to a higher GIB risk than warfarin (1.53% vs. 1.00% per year)^[^19^]^. Factors associated with increased GIB risk include older age, anemia, history of GIB, and long-term aspirin use^[^21,22^]^. While prior GIB increased the risk of subsequent major GIB, it did not affect stroke or mortality risk^[^22^]^. GIB in AF hospitalizations was associated with higher mortality, resource utilization, and costs^[^20^]^.

Notably, the burden of AF extends beyond cardiovascular events; it is associated with reduced quality of life and increased risk of cognitive decline, particularly in patients with silent cerebral infarcts and microbleeds^[^23^]^. These noncardiac outcomes highlight the need for comprehensive management strategies^[^24^]^. Emerging data suggest that socioeconomic status and racial disparities may influence AF detection and management, with underserved populations experiencing delayed diagnosis and limited access to NOACs^[^25^]^. Addressing these inequities is essential to improve outcomes on a population level^[^26^]^.

## Pathophysiological mechanisms

AF is associated with a hypercoagulable state and an increased risk of thromboembolism. This prothrombotic environment involves multiple factors fulfilling Virchow’s triad for thrombogenesis^[^27^]^. Patients with AF exhibit elevated coagulation markers, platelet activation, and endothelial dysfunction^[^28^]^. Evidence suggests a bidirectional relationship, where hypercoagulability may promote AF development, and AF itself exacerbates prothrombotic conditions^[^29^]^. Elevated vascular endothelial growth factor and tissue factor levels in AF patients further support the contribution of endothelial and vascular factors to this hypercoagulable state^[^30^]^. Additionally, AF-induced atrial remodeling may promote venous stasis, amplifying the risk of thromboembolic events^[^28^]^. Understanding these mechanisms is crucial for optimizing management strategies aimed at reducing thromboembolic complications in AF patients^[^27^]^.

Anticoagulant medications, including warfarin and antiplatelet agents such as aspirin, can increase GIB risk through mucosal injury and impaired hemostasis. Aspirin inhibits gastric prostaglandin synthesis, increasing both spontaneous and biopsy-induced bleeding, even when enteric-coated formulations are used^[^31^]^. Warfarin-associated GIB often occurs in patients with preexisting mucosal disease, most commonly peptic ulcers, which can be managed with endoscopic interventions^[^32^]^. The risk of UGIB is further heightened when low-dose aspirin is combined with nonsteroidal anti-inflammatory drugs (NSAIDs) or other anticoagulants, particularly during early therapy^[^33^]^. In patients with cerebrovascular disorders, stress-induced gastric mucosal damage is common and can be managed with acid suppression; however, long-term anticoagulant use may still increase susceptibility to drug-induced GI lesions^[^34^]^.

AF patients on anticoagulation therapy are at increased risk of GIB from diverse sources, including peptic ulcer disease, angiodysplasia, colonic diverticula, and GI malignancies. Peptic ulcers may be worsened by anticoagulants, *H. pylori* infection, or NSAID use. Angiodysplasia, particularly in elderly patients, is prone to rupture, while colonic diverticula frequently cause lower GIB. Malignancies, such as gastric and colorectal cancers, may result in occult or overt hemorrhage. Careful management of anticoagulation is therefore essential to balance stroke prevention with bleeding risk.

Recent evidence indicates a significant role for the gut microbiome in AF pathophysiology. Population-based studies have identified specific microbial genera associated with both prevalent and incident AF, including *Enorma* and *Bifidobacterium*^[^35^]^. Gut dysbiosis in AF patients is characterized by increased microbial diversity, overgrowth of *Ruminococcus, Streptococcus*, and *Enterococcus*, and reduced abundance of *Faecalibacterium, Alistipes, Oscillibacter*, and *Bilophila*^[^36^]^. These alterations are linked to changes in microbial metabolic activity, potentially influencing atrial electrophysiology and arrhythmogenesis^[^37^]^. Mechanistically, microbial metabolites such as trimethylamine N-oxide may promote a prothrombotic state and contribute to vascular dysfunction, connecting dysbiosis, thromboembolism, and GIB^[^38^]^. Translational implications include the potential use of probiotics, dietary modifications, and microbiome-targeted therapies for risk stratification and management of AF-related complications^[^37,39^]^.

Systemic inflammation in AF also contributes to GI vulnerability. Elevated cytokines, including interleukin-6 and tumor necrosis factor-alpha, are implicated in atrial remodeling and mucosal barrier dysfunction, increasing susceptibility to bleeding^[^40–42^]^. The gut–heart axis highlights how microbial metabolites can affect autonomic tone, atrial electrophysiology, and drug metabolism, further complicating anticoagulation management^[^43^]^. These findings underscore the potential of anti-inflammatory and gut-focused strategies in mitigating both AF progression and GI complications. The pathophysiological mechanisms of AF and GIB are summarized in Table 1.

**Table 1:** Pathophysiological mechanisms of AF and GI bleeding.

Mechanism	Causal link between AF and GI bleeding
Anticoagulant-induced GI mucosal injury	Anticoagulation, a cornerstone of AF management, directly increases the risk of GI bleeding by impairing hemostasis. Aspirin inhibits gastric prostaglandins, while warfarin exacerbates mucosal vulnerability.
Peptic ulcer disease in anticoagulated patients	AF patients on long-term anticoagulation are prone to peptic ulcers, especially when combined with NSAID use or *H. pylori* infection, leading to upper GI bleeding.
Angiodysplasia and age-related vascular fragility	Common in elderly AF patients, these fragile vascular malformations are more likely to bleed under anticoagulation.
Colonic diverticulosis	Frequently found in AF patients, especially older adults; anticoagulant therapy increases the risk of diverticular bleeding.
Gastrointestinal malignancies	Occult GI cancers may present with bleeding, which can be unmasked or worsened by anticoagulation therapy in AF patients.
Gut microbiome alterations and AF progression	Dysbiosis observed in AF patients (e.g., increased *Ruminococcus, Streptococcus*) may influence systemic inflammation and mucosal integrity, indirectly contributing to bleeding risks.

Furthermore, intestinal permeability and the concept of a “leaky gut” have been increasingly studied in the context of AF. Altered gut barrier function may allow translocation of microbial products such as lipopolysaccharides into the systemic circulation, triggering immune responses and promoting atrial fibrosis^[^44^]^. These findings support a novel paradigm where gut health and systemic inflammation are intertwined with AF pathophysiology, opening avenues for microbiome-targeted therapies.

## Anticoagulation and GIB risk

In patients with preexisting GI disorders, *personalized anticoagulation strategies* are essential. Individuals with a history of peptic ulcers, IBD, or other mucosal vulnerability may benefit from DOACs with relatively lower GIB risk profiles, such as apixaban, compared to rivaroxaban or dabigatran^[^11^]^. Proton pump inhibitors (PPIs) are recommended for gastroprotection in high-risk patients, particularly when concomitant antiplatelet therapy is used^[^12^]^. Multidisciplinary management, involving cardiology, gastroenterology, and primary care, is critical to optimize safety and clinical outcomes^[^45^]^.

1. **Risk stratification tools**

Several clinical risk scores have been evaluated for predicting GIB in patients receiving anticoagulants. While the CHA_2_DS_2_-VASc score was originally designed to estimate stroke risk, it has shown comparable performance to bleeding-specific tools such as HAS-BLED, ORBIT, and ATRIA in predicting NOAC-associated bleeding^[^13^]^. The predictive accuracy of these scores can vary depending on patient characteristics, including age, renal function, and concomitant antiplatelet therapy^[^41^]^. The HAS-BLED score remains particularly useful across different anticoagulant regimens and can be used in combination with CHA_2_DS_2_-VASc to balance thromboembolic benefits against bleeding risks^[^42^]^. Despite their utility, these tools demonstrate only modest performance, emphasizing the need for individualized assessment of bleeding risk factors^[^13,44^]^. Incorporating biomarkers such as hemoglobin levels, renal function (estimated Glomerular filtration rate), and prior bleeding history into risk algorithms may enhance predictive accuracy for GIB^[^46^]^. *A multimodal approach*, integrating clinical scores with laboratory data, can facilitate more personalized anticoagulation decisions^[^47^]^. In addition to traditionally used AF-specific risk models such as CHA_2_DS_2_-VASc, HAS-BLED, and ORBIT, contemporary cardiovascular research increasingly incorporates sophisticated, data-driven risk-stratification tools. For example, Oraii *et al* developed a retrospective cohort–based mortality risk model for patients with ST-elevation myocardial infarction undergoing primary percutaneous coronary intervention, demonstrating the expanding role of predictive analytics in cardiovascular outcome assessment and the evolution of risk-modelling methodologies across diverse clinical contexts^[^6^]^.

**2.Comparative risk of different anticoagulants**

DOACs and warfarin exhibit distinct patterns of GIB and ICH^[^11^]^.

- *Warfarin* is associated with a higher risk of *UGIB*, particularly in patients with underlying mucosal lesions, while rates of *lower GIB* are generally similar to those observed with DOACs^[^11^]^.
- Among DOACs, *rivaroxaban* carries the highest overall risk of GIB, whereas *apixaban* demonstrates the most favorable GI safety profile^[^11,12^]^. *Dabigatran* appears to have a lower risk of UGIB than rivaroxaban^[^11^]^.
- The risk of GIB increases with *advanced age*, particularly in patients ≥75 years, across all DOACs^[^12^]^.

Regarding *ICH*, rivaroxaban has been shown to significantly reduce risk compared to warfarin in AF patients^[^23^]^. Although some studies report higher numerical GIB rates with rivaroxaban compared to warfarin, the difference is often *not statistically significant*, highlighting the need for individualized therapy based on patient risk factors^[^23^]^.

**3.Antiplatelet therapy considerations**

Dual antiplatelet therapy (DAPT), combining aspirin with a P2Y12 inhibitor, is essential for preventing ischemic events in cardiovascular disease^[^24,25^]^. Current guidelines recommend 6–12 months of DAPT after drug-eluting stent implantation, though the optimal duration remains patient-specific^[^24^]^. Shorter DAPT courses (1–6 months) may be suitable for high-bleeding-risk patients, whereas extended therapy beyond 12 months reduces thrombotic events but increases bleeding risk^[^25^]^.

Triple antithrombotic therapy (DAPT plus anticoagulation) may be indicated for certain high-risk patients but carries a substantial bleeding hazard^[^26^]^. The selection and duration of antiplatelet therapy should be *individualized*, balancing efficacy against bleeding risk^[^25,40^]^. Ongoing research aims to optimize these strategies for different patient populations, integrating thrombotic and bleeding risk considerations^[^24,25^]^. Table 2 summarizes the comparative risk of anticoagulants and antiplatelet therapies.

**Table 2:** Comparative risk of anticoagulants and antiplatelet therapies.

Category	Warfarin	DOACs (apixaban, rivaroxaban, dabigatran, etc.)	Antiplatelet therapy (DAPT/triple therapy)
GI bleeding risk	Higher risk of upper GI bleeding than DOACs; similar risk for lower GI bleeding. In one study, GI bleeding incidence with warfarin was 2.0 per 100 patient-years (pt-yrs)^[^21^]^.	Rivaroxaban has the highest GI bleeding risk among DOACs (3.2/100 pt-yrs); apixaban has the lowest (1.6/100 pt-yrs). Dabigatran’s upper GI bleeding risk is lower than rivaroxaban^[^21,22^]^.	Increased GI bleeding risk especially when combined with aspirin + NSAIDs or anticoagulants. DAPT increases GI bleed risk by 2–3× compared to monotherapy^[^10,19,20^]^.
Intracranial hemorrhage (ICH) risk	Warfarin associated with higher ICH risk (~0.75/100 pt-yrs)^[^21^]^.	DOACs significantly reduce ICH risk vs. warfarin: Apixaban (0.33/100 pt-yrs), rivaroxaban (0.5/100 pt-yrs)^[^21,22^]^.	Triple therapy increases ICH risk significantly (up to 3× compared to dual therapy)^[^10,19^]^.
Age-related risk	GI bleeding risk increases with age; age >75 is an independent risk factor^[^20^]^.	Higher GI bleeding risk in elderly (≥75 years), especially with rivaroxaban; apixaban relatively safer in this group^[^20,22^]^.	Older patients on DAPT or triple therapy face elevated bleeding risks due to comorbidities and polypharmacy^[^10,19^]^.
Treatment duration considerations	Requires international normalized ratio (INR) monitoring; dietary restrictions (e.g., vitamin K). Typically used long-term in nonvalvular atrial fibrillation^[^6,7^]^.	No INR monitoring; fixed dosing but may need renal dose adjustment. Preferred in patients with poor INR control on warfarin^[^6,7,22^]^.	DAPT recommended for 6–12 months post-stenting. Shorter duration (1–6 months) in high-bleeding-risk patients; >12 months gives better ischemic protection but increases bleeding risk^[^10,19^]^.
Risk–benefit trade-off	High bleeding risk but proven efficacy in stroke prevention; narrow therapeutic index requires close monitoring^[^6,7^]^.	Comparable or superior efficacy with reduced ICH risk. GI bleeding risk varies across agents. Apixaban often preferred for better safety profile^[^21,22^]^.	Essential for reducing stent thrombosis and recurrent ischemia, but must be balanced against major bleeding risk, especially in elderly or those with prior bleeds^[^10,19,20^]^.

## Management strategies

1. **Risk mitigation approaches**

AF significantly increases stroke risk, necessitating careful anticoagulation management. Patient selection based on established risk criteria is critical to optimize outcomes while minimizing bleeding risk^[^38^]^. Clinical decision aids that incorporate both stroke and bleeding risk assessments can guide anticoagulant selection, demonstrating utility in managed care settings^[^43^]^. The CHA_2_DS_2_-VASc score helps identify candidates for non-vitamin K oral anticoagulants (NOACs), which are generally preferred over warfarin due to favorable safety and efficacy profiles^[^46^]^. Heterogeneity among AF patients underscores the need for individualized strategies tailored to specific comorbidities, bleeding risk, and patient characteristics^[^47^]^. Collectively, these approaches advocate for a nuanced, patient-centered model for anticoagulation in AF.

PPIs reduce the risk of UGIB in AF patients on DOACs^[^48–50^]^. The protective effect is most pronounced in high-risk patients, including those aged ≥75 years, with HAS-BLED scores ≥3, prior GIB history, or concurrent antiplatelet therapy^[^48,49^]^. Even low-dose PPI therapy significantly lowers the risk of major GIB^[^50^]^. For high-risk AF patients, *pretreatment endoscopic evaluation* may identify mucosal lesions such as erosive gastritis, ulcers, or esophageal varices, which could influence anticoagulant choice and gastroprotective strategies^[^51^]^.

**2.Acute management of GIB in AF patients**

The acute management of GIB in AF patients requires rapid assessment and stabilization. Initial measures include *hemodynamic support* with fluids and blood transfusions as indicated^[^52^]^. Anticoagulants and antiplatelets are temporarily withheld, with *reversal agents considered in life-threatening bleeding*^[^10^]^. Endoscopy is the primary modality for identifying bleeding sources and assessing severity, with colonoscopy recommended within 24 h for lower GIB^[^53^]^. PPIs can stabilize mucosal clots and support hemostasis^[^52^]^.

When restarting anticoagulation, timing must balance bleeding and thrombotic risk^[^10^]^. Non-vitamin K oral anticoagulants (NOACs) are often preferred over warfarin due to lower overall bleeding risk, particularly with *apixaban*, which demonstrates the most favorable GI safety profile^[^54^]^. Chronic kidney disease and warfarin use have been associated with increased mortality in AF patients with GIB^[^54^]^.

Interventional radiology is a key adjunct when endoscopic hemostasis fails or is contraindicated^[^55–57^]^. Imaging modalities such as *CT angiography* and *nuclear scintigraphy* help localize bleeding sources and guide therapeutic interventions^[^56^]^. *Transcatheter angiography* and *embolization* are effective for achieving hemostasis in refractory cases^[^55,56^]^. The role of interventional radiology has expanded due to an aging population and widespread anticoagulant use, offering prompt and effective management for severe or persistent GIB^[^58^]^.

The development of *specific reversal agents* has further enhanced NOAC safety. Idarucizumab targets dabigatran, and andexanet alfa targets factor Xa inhibitors, indicated for life-threatening bleeding or urgent surgery^[^59–61^]^. Prothrombin complex concentrate remains an option for warfarin reversal^[^62^]^. Universal reversal agents, such as ciraparantag, are under FDA Fast-Track Review^[^62^]^. Despite their benefits, cost and limited availability remain considerations^[^61^]^.

**3.Resumption of anticoagulation post-GIB**

Resuming anticoagulation after GIB is generally beneficial but requires careful timing^[^63,64^]^. Restarting therapy reduces thromboembolic events and improves outcomes^[^63,64^]^. Evidence suggests the optimal window for resumption is *7–30 days post-GIB*, balancing recurrent bleeding against thromboembolism risk^[^63–65^]^. Some studies propose *30–50 days* in specific high-risk populations^[^66^]^. Decisions should be individualized, incorporating patient comorbidities, bleeding etiology, and overall risk profile^[^63,66^]^.

For DOACs, *apixaban* is often preferred due to its lower GIB risk^[^63^]^. The decision to restart anticoagulation should involve shared decision-making, weighing benefits, potential harm, and patient preferences^[^64,66^]^.

Table 3 summarizes the management strategies for anticoagulation in AF patients with GIB.

**Table 3:** Management strategies for anticoagulation in AF patients with GI bleeding.

Management strategy	Key considerations	Recommendations
Risk mitigation approaches	AF increases stroke risk, requiring careful anticoagulation management.	- Use CHA_2_DS_2_-VASc and HAS-BLED scores for patient selection.
		- NOACs preferred over warfarin due to better safety profile.
		- Individualized treatment based on patient risk factors.
Gastroprotection strategies	AF patients on DOACs have a risk of upper GI bleeding.	- Proton pump inhibitors (PPIs) reduce upper GI bleeding risk.
		- Most beneficial in high-risk patients (≥75 years, HAS-BLED ≥3, prior GI bleeding, or on antiplatelet therapy).
		- Pretreatment endoscopic evaluation recommended in high-risk cases.
Acute management of GIB	Requires rapid assessment and intervention.	- Stabilize hemodynamics with fluid resuscitation and blood transfusions.
		- Temporarily stop anticoagulants & consider reversal for life-threatening bleeding.
		- Endoscopy is the first-line diagnostic tool; colonoscopy within 24 h for lower GI bleeding.
		- Use PPIs to stabilize mucosal clots.
Interventional radiology	Used when endoscopic treatment fails or is contraindicated.	- Diagnostic imaging (CT angiography, nuclear scintigraphy) to localize bleeding.
		- Transcatheter angiography & embolization for refractory cases.
		- Increasingly important due to aging population & rising anticoagulant use.
Reversal of anticoagulation	Reversal agents are crucial for life-threatening bleeding or emergency surgery.	- Idarucizumab (for dabigatran) and andexanet alfa (for factor Xa inhibitors) approved by FDA.
		- Prothrombin complex concentrate used for warfarin reversal.
		- Ciraparantag, a universal reversal agent for all NOACs, is under FDA Fast-Track Review.
Resumption of anticoagulation post-GIB	Balancing stroke prevention and re-bleeding risk is critical.	- Restarting anticoagulation reduces thromboembolism risk.
		- Optimal resumption timing: 7–30 days post-GIB; some studies suggest 30–50 days.
		- Apixaban preferred due to lower GI bleeding risk.
		- Decision should involve shared decision-making with the patient.

## Future directions and unanswered questions

Future research should focus on developing novel anticoagulants that minimize the risk of GIB while maintaining effective stroke prevention in AF patients. Identifying biomarkers and genetic predictors of bleeding risk could enhance personalized medicine approaches, allowing for more precise risk stratification and individualized treatment plans. Additionally, AI-driven predictive models have the potential to revolutionize anticoagulation management by integrating patient-specific data to optimize therapy selection and dosing. Emerging evidence suggests that gut microbiome alterations may play a role in AF pathophysiology, warranting further investigation into microbiome-targeted interventions as a potential therapeutic avenue. Ongoing clinical trials are also exploring alternative stroke prevention strategies for patients at high risk of bleeding, which could lead to safer and more effective treatment paradigms.

## Conclusion

AF is a major risk factor for thromboembolism, necessitating anticoagulation therapy, which unfortunately increases the risk of GIB. Effective management of this complex interplay requires robust risk stratification tools, careful selection of anticoagulants, and evidence-based strategies to mitigate bleeding complications. Multidisciplinary collaboration among cardiologists, gastroenterologists, and hematologists is essential to optimize patient outcomes. Future research should aim to refine anticoagulation strategies, improve predictive modeling for bleeding and thromboembolism risk, and explore novel treatment approaches that enhance both safety and efficacy in AF management.

## Data Availability

Not applicable for this review.
